# Unplanned hemodialysis initiation and low geriatric nutritional risk index scores are associated with end-stage renal disease outcomes

**DOI:** 10.1038/s41598-022-14123-y

**Published:** 2022-06-30

**Authors:** Ryoichi Maenosono, Tatsuo Fukushima, Daisuke Kobayashi, Tomohisa Matsunaga, Yusuke Yano, Shunri Taniguchi, Yuya Fujiwara, Kazumasa Komura, Hirofumi Uehara, Maki Kagitani, Hajime Hirano, Teruo Inamoto, Hayahito Nomi, Haruhito Azuma

**Affiliations:** 1 Department of Urology, Osaka Medical and Pharmaceutical University, 2-7 Daigaku-machi, Takatsuki, Osaka 569-8686 Japan; 2 Department of Urology, Hirakata City Hospital, 2-14-1 Kinya-honmachi, Hirakata, Osaka 573-1013 Japan; 3 Department of Urology, Matsubara Tokushukai Hospital, 7-13-26 Amami-higashi, Matsubara, Osaka 580-0032 Japan; 4 Blood Purification Center, Osaka Medical and Pharmaceutical University Hospital, 2-7 Daigaku-machi, Takatsuki, Osaka 569-8686 Japan

**Keywords:** Continuous renal replacement therapy, Haemodialysis, Nutrition

## Abstract

Patients with end-stage renal disease (ESRD) have a low nutritional status and a high mortality risk. The geriatric nutritional risk index (GNRI) is a predictive marker of malnutrition. However, the association between unplanned hemodialysis (HD) and GNRI with mortality remains unclear. In total, 162 patients underwent HD at our hospital. They were divided into two groups: those with unplanned initiation with a central venous catheter (CVC; n = 62) and those with planned initiation with prepared vascular access (n = 100). There were no significant differences in sex, age, malignant tumor, hypertension, and vascular disease, while there were significant differences in the times from the first visit to HD initiation (zero vs. six times, p < 0.001) and days between the first visit and HD initiation (5 vs. 175 days, p < 0.001). The CVC insertion group had significantly lower GNRI scores at initiation (85.7 vs. 99.0, p < 0.001). The adjusted hazard ratios were 4.002 and 3.018 for the GNRI scores and frequency, respectively. The 3-year survival rate was significantly lower in the CVC + low GNRI group (p < 0.0001). The GNRI after 1 month was significantly inferior in the CVC insertion group. Inadequate general management due to late referral to the nephrology department is a risk factor for patients with ESRD.

## Introduction

The number of patients with end-stage renal disease (ESRD) requiring renal replacement therapy (RRT) is increasing worldwide^1,2^. As the elderly population increases, a higher proportion of frail patients needing dialysis is emerging globally. Although the development of hemodialysis (HD) management helps prolong patients’ lifespans, it is well known that inadequate preparation, such as an unplanned HD initiation that needs emergent central venous catheter (CVC) insertion affects mortality^3^. Compared to arteriovenous fistulas, CVC insertion has a hazard ratio (HR) of 1.53 (95% confidence interval [CI] 1.41–1.67) for all-cause mortality^4^; hence, HD with CVC is considered a risk factor for mortality. The arteriovenous fistulas should be created at 4 weeks before the scheduled HD initiation, as it takes at least 2 weeks for maturation^5^. This suggests that patients with chronic kidney disease (CKD) should be referred to the nephrology department at the appropriate time, with the access prepared before initiation of dialysis initiation^6^, since late referral to nephrologists could lead to unfavorable outcomes^7–9^.

In contrast, patients with ESRD and uremia can develop malnutrition^10^, with a prevalence of 28–54% in patients undergoing HD^11^. Wei et al. showed that malnutrition could be associated with frailty, a geriatric syndrome that reflects multisystem physiological dysregulation^12^. Indeed, malnutrition in patients with ESRD and prefrailty/frailty was reported to be a significant risk factor for poor outcomes^11,12^.

Several prediction scores for the mortality of malnourished patients with ESRD have been investigated broadly. Biochemical indices, such as serum albumin, C-reactive protein, and ferritin levels, are well-known prediction markers. In contrast, the physical index described by a subjective global assessment, subjective global assessment-dialysis malnutrition, malnutrition inflammation, and geriatric nutrition risk index (GNRI) scores have also been studied and can be used to evaluate the nutritional status^13–16^. GNRI was originally developed by Bouillanne et al.^17^ for identifying geriatric hospitalized patients with a nutritional risk and is based on simple indices, such as serum albumin levels and patient’s bodyweight. To date, several studies have shown that the GNRI, as one of the prognostic indices, can be used to predict the mortality of frail patients with CKD undergoing HD^7,8,18^.

GNRI at the time of initiation of dialysis is valuable for predicting mortality (GNRI low, 22.2% vs. GNRI high, 12.6%, p < 0.001)^19^. Although many studies have demonstrated the effectiveness of the GNRI as a predictive marker in the management of CKD or maintenance of patients with frailty and malnutrition undergoing HD, only a few have described whether the GNRI scores at HD initiation can impact the outcome of unplanned HD initiation following inadequate preparation due to late referral. Thus, we investigated whether the association between unplanned dialysis and nutritional status affected patient outcomes.

## Results

### Baseline characteristics

There was no significant between-group difference regarding sex, age, primary illness (such as active malignant tumors, diabetes mellitus, and hypertension), and past medical history, including ischemic heart disease, neurovascular disease, and peripheral artery disease (Table 1). Visit frequency until HD initiation was required for ESRD was significantly lower in the CVC insertion group than in the vascular access initiation group (zero vs. six times, respectively; p < 0.001). Similarly, the association of the number of days between the first visit and HD initiation (5 vs. 175 days, respectively; p < 0.001) and that between vascular access placement and HD were significantly lower in the CVC insertion than that in the vascular access initiation group (− 17 vs. 70 days, respectively; p < 0.001; the minus sign indicates that vascular access was created after HD initiation). Interestingly, there was a statistically significant between-group difference in the GNRI scores at HD initiation (CVC insertion vs. vascular access, 85.7 vs. 99.0%; p < 0.001), which was similar to the visit frequency. Unplanned HD with CVC insertion and a low GNRI score were considered consequences of late referral to the nephrology department.

**Table 1:** Clinical characteristics of patients undergoing HD.

	CVC insertion (n = 62)	Vascular access (n = 100)	p-value
Male, number (%)	39 (62.9%)	67 (67.0%)	0.284
Age [IQR]	72 [65, 79]	71 [60, 79]	0.878
**Visit frequency until HD initiation**	0 [0, 2]	6 [4, 11]	< 0.001
Days between first visit and HD initiation	5 [0, 43]	175 [87, 367]	< 0.001
Days between VA preparation and HD initiation	− 17 [− 27, − 7]	70 [20, 143]	< 0.001
GNRI at HD initiation [IQR]	85.7 [80.3, 99.0]	99.0 [94.1, 106.4]	< 0.001
Adjusted calcium (mg/dL)	8.8 ± 1.0	8.7 ± 0.8	0.446
Inorganic phosphorus (mg/dL)	6.5 ± 2.8	6.0 ± 1.2	0.260
Intact PTH (pg/mL)	256.3 ± 23.4	239.3 ± 18.1	0.566
Hemoglobin (g/dL)	8.6 ± 1.8	9.7 ± 1.6	< 0.001
Malignant tumor, number (%)	13 (21.0%)	12 (12.0%)	0.179
Diabetes, number (%)	29 (46.8%)	43 (43.0%)	0.745
Hypertension, number (%)	44 (71.0%)	77 (77.0%)	0.458
PAD, number (%)	6 (9.7%)	12 (12.0%)	0.799
PMH, ischemic heart disease, number (%)	12 (19.4%)	22 (22.0%)	0.843
PMH, neurovascular disease, number (%)	6 (9.7%)	12 (12.0%)	0.799
eGFR at initiation, mean	6.3 ± 3.4	5.4 ± 1.8	0.145

CVC, central venous catheter; eGFR, estimated glomerular filtration rate; GNRI, geriatric nutritional risk index; HD, hemodialysis; IQR, interquartile range; PAD, peripheral artery disease; PMH, past medical history; PTH, parathyroid hormone; RRT, renal replacement therapy; VA, vascular access.

### Risk factors of patients’ outcomes on time of HD initiation

**Figure 1:**
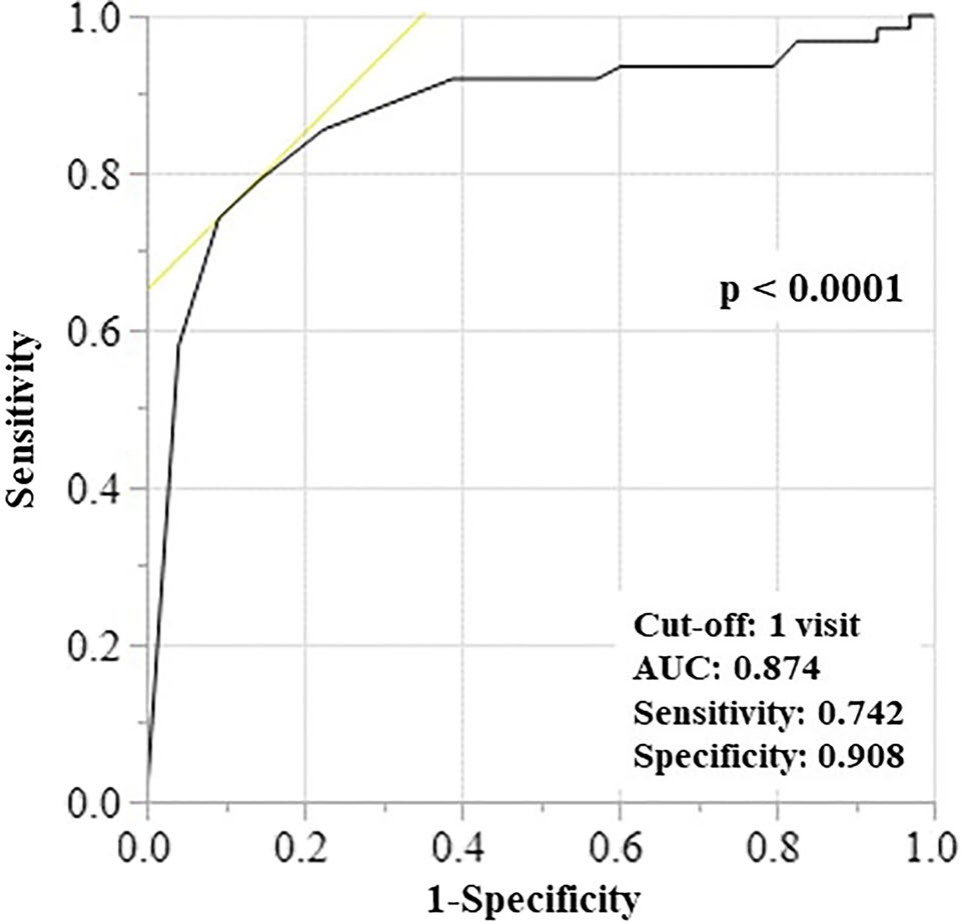
ROC analysis based on the visit time for CVC insertion at HD initiation. In this model, the cut-off value of visit time is 1; sensitivity and specificity were 74.2% and 90.8%, respectively; the AUC was 0.874, p < 0.0001. AUC, area under the curve; CVC, central venous catheter; HD, hemodialysis; ROC, receiver operating characteristic.

Next, we analyzed the relationship between visit frequency and the incidence of CVC insertion using an established receiver operating characteristic (ROC) curve (area under the curve, 0.874; p < 0.0001), with a value of 1 (one visit), which indicated the highest sensitivity and specificity (Fig. 1). Univariate Cox regression analysis showed that GNRI (low vs. high) at HD initiation and the visit frequency (≤ 1) were significant risk factors for poor outcomes (hazard ratio [HR], 3.781; 95% confidence interval [CI], 1.766–8.096; p = 0.001 vs. HR, 4.064; 95% CI 1.809–9.130; p = 0.001), in addition to age (> 75 years old) and brain/cardiovascular disorders. Although age and brain/cardiovascular disorders are recognized as important factors for patients’ outcomes in patients with CKD (HR, 2.930; 95% CI 1.361–6.310; p = 0.006 vs. HR, 2.402; 95% CI 1.125–5.129; p = 0.025), the multivariate-adjusted Cox regression analysis also indicated that the GNRI scores and visit frequency were also significant risk factors for poor outcomes (HR, 4.002; 95% CI 1.608–9.964; p = 0.003 vs. HR, 3.018; 95% CI 1.258–7.239; p = 0.011; Table 2). Thus, these analyses revealed that the nutritional status and visit frequency also had a critical impact on survival outcomes.

**Table 2:** Risk factors affecting patient survival after HD initiation.

	Univariate			Multivariate^a^		
	Hazard ratio	95% CI	p-value	Hazard ratio	95% CI	p-value
Sex, male	0.856	[0.397, 1.847]	0.692			
Age, > 75 years	2.930	[1.361, 6.310]	0.006	3.900	[1.724, 8.823]	0.001
Brain/cardiovascular disorder	2.402	[1.125, 5.129]	0.025	3.062	[1.325, 7.077]	0.004
GNRI*, low vs. high	3.781	[1.766, 8.096]	0.001	4.002	[1.608, 9.964]	0.003
Hemoglobin ≥ 10 (g/dL)	0.507	[0.205, 1.257]	0.121			
Visit frequency ≤ 1	4.064	[1.809, 9.130]	0.001	3.018	[1.258, 7.239]	0.011

*The GNRI score was calculated using the following formula: GNRI = (14.89 × sAlb) + 41.7 × (BW/IBW).

sAlb, serum albumin; BW, bodyweight; CI, confidence interval; GNRI, geriatric nutritional risk index; HD, hemodialysis; IBW, ideal body weight.

^a^The multivariate Cox regression model initially included age, brain and cardiovascular disorder, GNRI score, and visit frequency.

### Association of unplanned HD initiation and malnutrition prior to initiation with patient mortality

**Figure 2:**
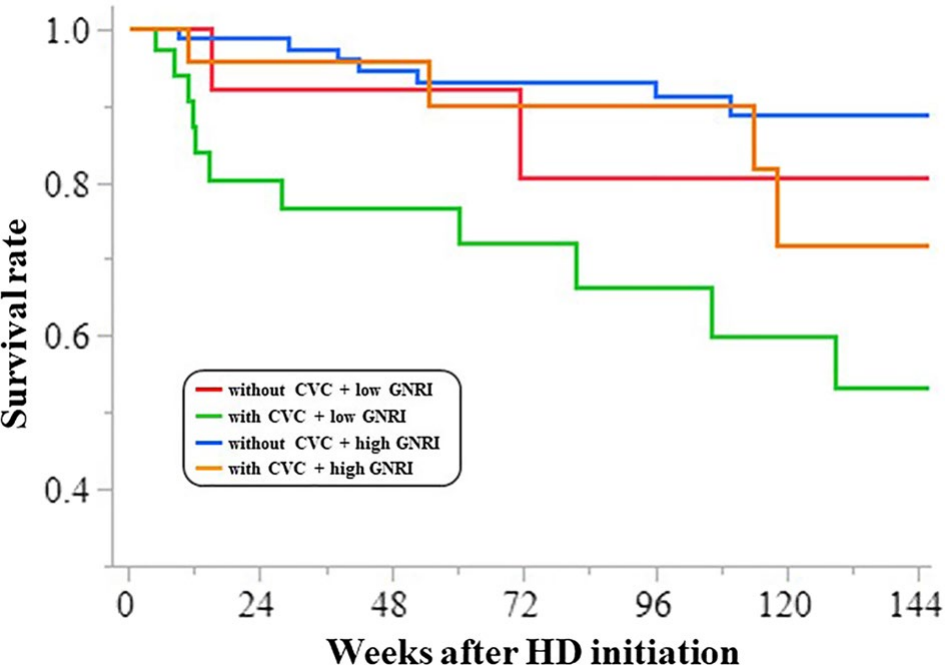
The 3-year survival rates after HD initiation. We compared four groups based on two parameters: with CVC + low GNRI score, without CVC + low GNRI score, without CVC + high GNRI score, and with CVC + high GNRI score. Those who belonged to the group “with CVC + low GNRI” had the lowest survival rate (46.5%, p < 0.0001) compared to the other groups (80.9%, 88.9%, and 71.6%, respectively). CVC, central venous catheter; GNRI, geriatric nutritional risk index; HD, hemodialysis.

Next, we investigated whether the combination of malnutrition and HD initiation with CVC insertion could lower the all-cause mortality. Patients were divided into four groups according to their GNRI scores and CVC insertion: those with CVC + low GNRI score, those without CVC + low GNRI score, those without CVC + high GNRI score, and those with CVC + high GNRI score. Although the 3-year patient survival in the “with CVC + low GNRI” group was inferior (46.5%; p < 0.0001) as compared to the other groups, the remaining three groups were not significantly different (80.9, 88.9, and 71.6%; p = 0.5379; Fig. 2). Almost all the events occurred within 48 weeks of initiation.

**Figure 3:**
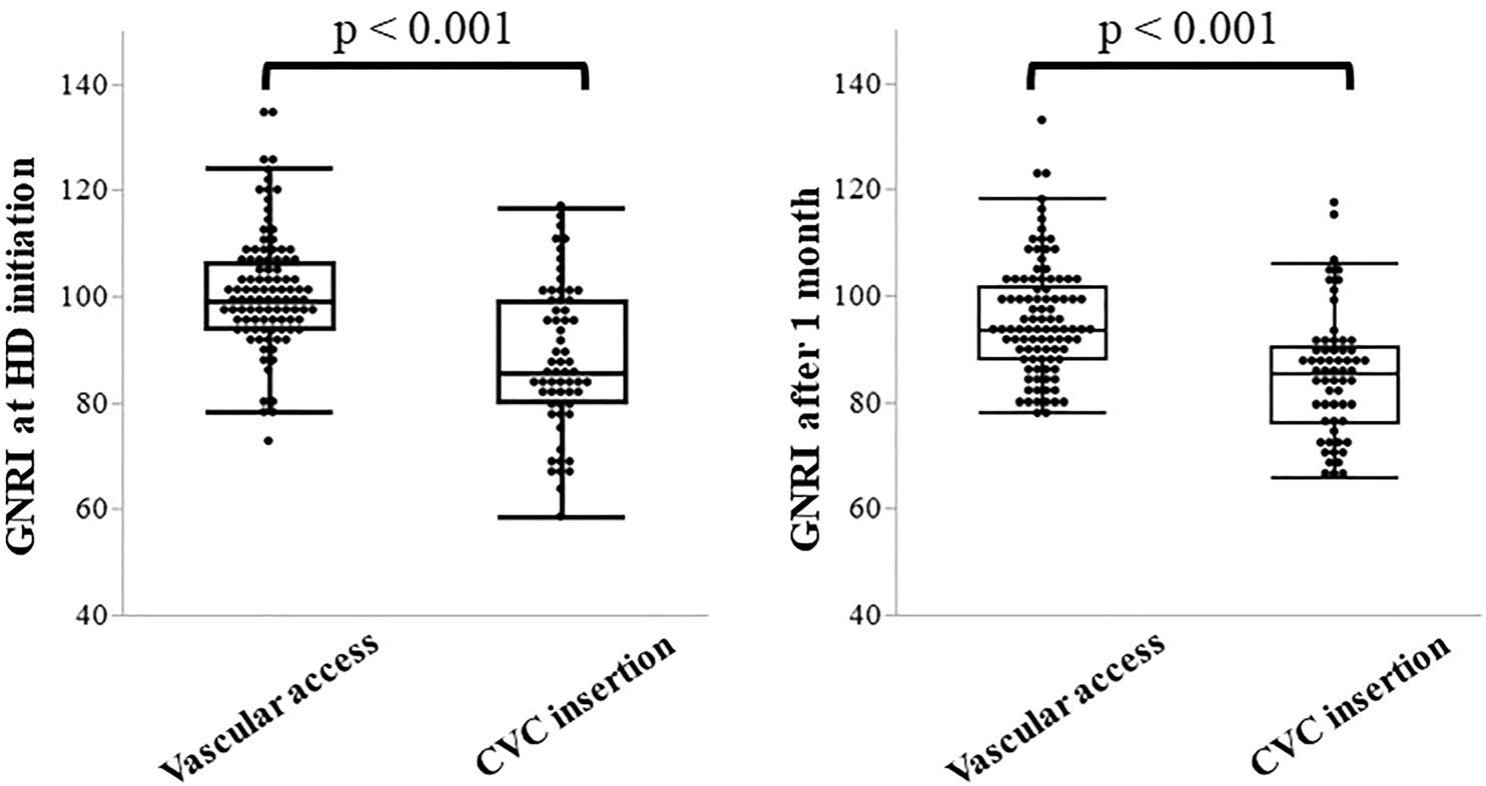
The transition of GNRI between HD initiation and 1 month after initiation. At the time of initiation, the GNRI score was significantly lower in the CVC group compared to that in the vascular access group. Similarly, at 1 month after initiation, the low value of GNRI persisted in the CVC insertion group. CVC, central venous catheter; GNRI, geriatric nutritional risk index; HD, hemodialysis.

Furthermore, we analyzed the transition of the GNRI scores after HD initiation between CVC insertion and vascular access groups. The GNRI score was significantly lower in the CVC insertion group (median, interquartile range: 85.7 [80.3–99.0] points) compared to the vascular access group (99.0, [94.1–106.4] points; p < 0.001), and remained significantly lower even after 1 month (85.3 [76.1–90.2 vs. 93.6 [88.2–101.7] points; p < 0.001), showing that low GNRI scores persisted despite HD initiation (Fig. 3).

## Discussion

We analyzed the association between malnutrition and inadequate preparation for HD and identified that unplanned HD initiation due to late referral to a nephrology department could lead to poor patient outcomes. In this retrospective cohort study, although there were no significant between-group differences regarding age, sex, primary illness, and past medical history, the visit frequency and GNRI scores were significantly different, which implied that patients who underwent CVC insertion did not have sufficient time for the preparation of a vascular access. Visit frequency ≤ 1 could be the cut-off value to denote CVC insertion and unplanned HD initiation. The 3-year patient survival rate was significantly lower in the group with CVC and low GNRI scores as compared to the other combination groups. Furthermore, the GNRI scores of the CVC group also had a significantly lower value despite HD treatment.

The number of patients with ESRD is increasing worldwide, and more elderly patients are undergoing dialysis^1^. As the number of frail patients with CKD is increasing, physicians should manage patients’ health and consider their preferred choice—if renal function decreases and patients wish to undergo RRT, they should be referred to experts^12^. In a 2019 update, the Kidney Disease Outcome Quality Initiative (KDOQI) recommended that the ESRD life-plan should be discussed with the patient within a multidisciplinary team framework. A nephrologist should at least discuss modality options with the patient, with referral to a vascular access surgeon for input on the appropriate dialysis access that corresponds to the chosen RRT modality^20^. Especially, vascular access should be prepared at an appropriate time to avoid the risk of emergent CVC insertion. The Japanese Society for Dialysis Therapy guidelines describe that vascular access construction should be considered for CKD stages 4 or 5 while considering clinical conditions, and an arteriovenous fistula should be constructed at least 2–4 weeks before the initial puncture^21^.

A previous study showed that an unplanned start of dialysis was associated with poor survival. Roy et al. demonstrated that the survival rates at 3 and 12 months were 38.6% vs. 90.9% and 14.4% vs. 73.6% for unplanned vs. planned dialysis, respectively (p < 0.001). These findings showed that insufficient preparation was a risk factor for these patients^3^. Indeed, immature vascular access due to late referral could lead to unplanned dialysis^22^. Conversely, elderly ESRD patients are less likely to undergo surgery for the creation of vascular access and have a higher likelihood of dying after HD initiation; hence, CVC itself could not be a risk factor for mortality in those populations^23,24^. However, there is no universal definition for referral timing in patients with CKD, and it varies across institutions^25–27^. Although the optimal cut-off value remains controversial, the most broadly accepted definition of late referral is the first encounter with an expert within 3–4 months prior to ESRD diagnosis^6,28^. Our clinical data also showed that the participants in the vascular access group visited a physician to consider RRT at approximately ≥ 3 months (175 [range, 87–365] days) prior to ESRD diagnosis requiring HD initiation.

Other reasons why physicians should refer patients with ESRD to a nephrology department are as follows: potential loss of appetite due to cytokine production, malabsorption due to gut edema, and difficulty in oral intake arising from general fatigue^29^. According to the 2020 KDOQI guidelines, CKD malnutrition care should be undertaken by multidisciplinary teams^30^. To evaluate the malnutrition status, GNRI was developed. Interestingly, previous studies have shown that it is useful for evaluating the mortality of patients with CKD^8,9,17,19^. One report stated that the GNRI was useful in predicting mortality in patients with CKD at the time of dialysis initiation^19^. Conversely, many studies have demonstrated that the GNRI could be an effective predictive marker in patients undergoing HD^7,8,31^. However, it remains unclear whether urgent HD initiation due to late referral and nutritional status is associated with patient outcomes. In our study, the emergent CVC insertion group had a lower visit frequency until HD initiation than the vascular access group and had lower GNRI scores, which has been recognized as a predictor of mortality. The fact that the CVC + low GNRI group had the lowest 3-year survival demonstrated that inappropriate patient evaluation or late referral to a nephrology department may lead to poor patient outcomes.

The major limitations of this study included the small patient cohort, the retrospective and short-term nature of the study, and the insufficient definition of the appropriate referral timing. Furthermore, factors that could not be measured and residual confounding factors might have affected our results. Indeed, we did not include tunneled-cuffed permanent CVC and compared the model performance of these nutritional indices with the malnutrition inflammation scores^14^, which is a standard nutritional assessment tool frequently used in patients undergoing HD. Additionally, we could not retrospectively examine the rationale behind the visit frequency affecting the GNRI scores. Further studies are needed to investigate the causes of the low number of visits in detail. GNRI is not only a nutritional score, but is also a well known surrogate marker for predicting the survival of patients with CKD^7,8,18^^.^. Therefore, a similar study has to be conducted among patients undergoing unplanned initiation. Finally, because patients undergoing HD visited different HD clinics as outpatients after discharge, we did not consider residual bias that could exist in each clinic’s management.

In conclusion, the combination of unplanned HD initiation with emergent CVC insertion and low GNRI scores with low nutritional status was significantly associated with poor patient outcomes. Although this study had some limitations, our results support the critical role of managing patients with CKD who require RRT during the preservation period. We strongly recommend that non-nephrology experts should refer such patients to the appropriate nephrology department to facilitate early management of CKD. However, patient outcomes in ESRD may not be strictly associated with one nutritional factor, and further studies should focus on a larger number of patients, with detailed nutritional information over longer follow-up periods.

## Methods

This study was approved by the Osaka Medical and Pharmaceutical University ethics committee in accordance with institutional guidelines. The study conformed to the guidelines of the Declaration of Helsinki. Written Informed consent for participation was obtained from all the participants involved in the study.

### Study population and clinical design

**Figure 4:**
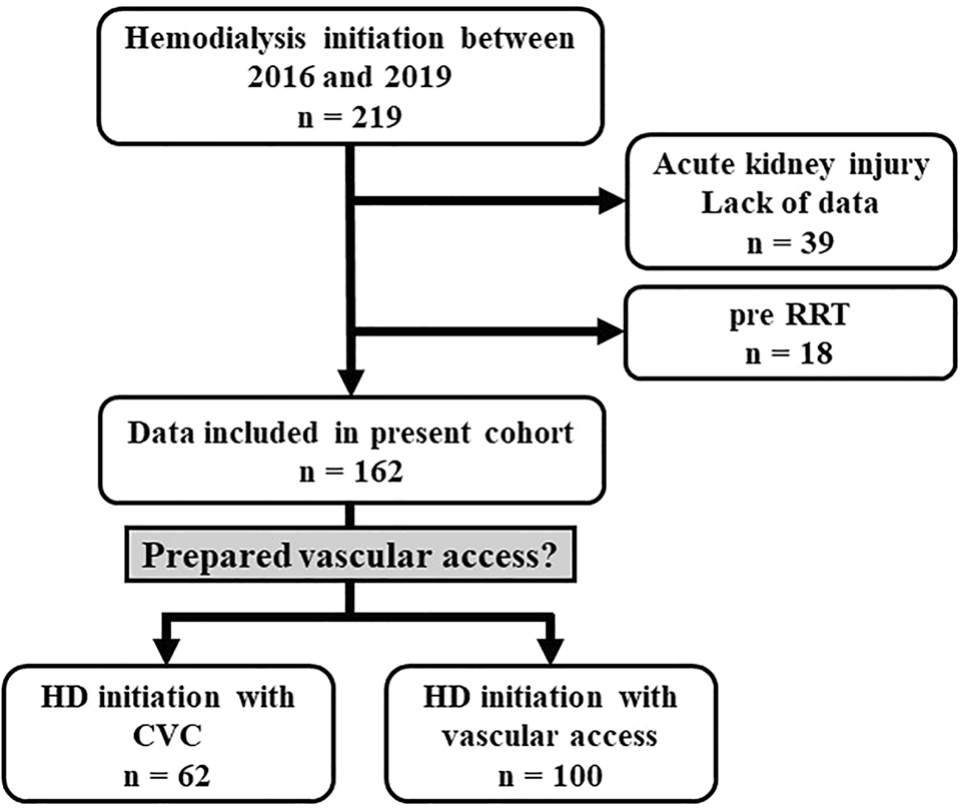
Flowchart of patient selection. Out of 219 patients screened between January 2016 and December 2019, we excluded 39 because of the presence of acute kidney injury and lack of data. Ordinarily, patients are referred to our department from other departments or clinics to prepare for vascular access and HD initiation. Of the 162 patients, the CVC and vascular access groups contained 62 and 100 patients, respectively. CVC, central venous catheter; HD, hemodialysis.

A total of 219 patients with ESRD, who had started HD at Osaka Medical and Pharmaceutical University Hospital between January 2016 and December 2019, were retrospectively enrolled in this study. These patients were referred to our department from clinics or other departments to consider RRT. Among these patients, 39 were excluded because of lack of clinical data or acute kidney injury, while 18 were also excluded because they had a medical history of pre-RRT. A total of 162 patients were finally included in this study and were divided into two groups based on whether HD initiation was emergent CVC insertion or prepared vascular access (Fig. 4).

In our institute, patients with CKD are generally referred to our department to select RRT options. In this cohort, the patients who underwent planned HD underwent vascular access surgery in our department, and HD was subsequently initiated depending on their general condition. Unplanned HD initiation was defined as dialysis initiation when access was not ready for use, patients required hospitalization, or dialysis was initiated with a modality that was not the one that was initially selected for the patient (e.g., CVC insertion)^32^. Here, CVC indicates that the patient needed emergent HD with a temporary non-cuffed double lumen catheter due to a progressed CKD condition. The first visit referred to the day on which patients had a consultation or were referred to our department, and the visit frequency was the time between the first visit and the first HD session.

### The geriatric nutrition risk index

The GNRI scores were calculated from the patients’ serum albumin value and bodyweight using the following formula: GNRI = (14.89 × serum albumin [mg/dL]) + 41.7 × (bodyweight/ideal bodyweight)^17^. The bodyweight was measured in kg at the time of HD initiation and then, 1 month later. The ideal bodyweight was calculated from the patient’s height and a body mass index of 22 kg/m^2^. The GNRI scores were used as both continuous and categorical variables. Here, we adopted a GNRI score of 91.2 points as the cut-off for dividing the low and high GNRI groups in accordance with a previous study^30,31^.

### Statistical analysis

Demographic information is summarized using frequency counts, means with standard deviations, or medians with interquartile ranges. The chi-square and Fisher’s exact tests were used to compare categorical variables, and Student’s t-test or one-way analysis of variance was used to compare continuous variables, as appropriate. ROC curve analysis was used to investigate whether visit frequency values could distinguish between CVC insertion and non-insertion at HD initiation. The frequency with the best accuracy was selected as the cut-off value. A multivariate Cox proportional hazard regression model was used to assess risk factors for all-cause mortality. The 3-year survival rate was initially estimated using Kaplan–Meier analysis. The population of kidney transplantation after HD initiation was considered censored in the survival analysis. Univariate analysis was used to examine prognostic factors for the final multivariate Cox regression analysis model. Factors that reached significance in the univariate analysis were subsequently included in the multivariate Cox model to determine the independent effects of each factor. Two-sided p-values < 0.05 were considered statistically significant. All the statistical analyses were performed using JMP Software 15 Pro version (SAS Institute, Cary, NC, USA).

## Data Availability

The data supporting this study's findings are available on request from the corresponding author (HA).
